# Effects of different Fe supplies on mineral partitioning and remobilization during the reproductive development of rice (*Oryza sativa* L.)

**DOI:** 10.1186/1939-8433-5-27

**Published:** 2012-09-28

**Authors:** Raul Antonio Sperotto, Marta Wilton Vasconcelos, Michael Andrew Grusak, Janette Palma Fett

**Affiliations:** Centro de Biotecnologia, Universidade Federal do Rio Grande do Sul, 91501-970 Porto Alegre, RS Brazil; Departamento de Botânica, Universidade Federal do Rio Grande do Sul, 91501-970 Porto Alegre, RS Brazil; Centro de Ciências Biológicas e da Saúde, Centro Universitário UNIVATES, 95900-000 Lajeado, RS Brazil; CBQF/Escola Superior de Biotecnologia, Universidade Católica Portuguesa, Rua Dr. António Bernardino de Almeida, 4200-072 Porto, Portugal; USDA/ARS Children’s Nutrition Research Center, Department of Pediatrics, Baylor College of Medicine, 1100 Bates Street, Houston, TX 77030 USA

**Keywords:** Biofortification, Elemental analysis, Ionomics, Iron (Fe), Kitaake, Mineral partitioning, Reproductive development of rice

## Abstract

**Background:**

Minimal information exists on whole-plant dynamics of mineral flow through rice plants and on the source tissues responsible for mineral export to developing seeds. Understanding these phenomena in a model plant could help in the development of nutritionally enhanced crop cultivars. A whole-plant accumulation study, using harvests during reproductive development under different Fe supplies, was conducted to characterize mineral accumulation in roots, non-flag leaves, flag leaves, stems/sheaths, and panicles of Kitaake rice plants.

**Results:**

Low Fe supply promoted higher accumulation of Zn, Cu and Ni in roots, Mn, Ca, Mg and K in leaves and Zn in stems/sheaths and a smaller accumulation of Fe, Mn and Ca in roots and Zn and Ni in leaves. High Fe supply promoted higher accumulation of Fe in roots and Zn in leaves and a smaller accumulation of Fe in leaves and stems/sheaths and Zn, Cu and K in roots. Correlation analyzes indicated that fluctuations in Mn-Ca, Zn-Cu, Zn-Ni, Cu-Ni, Mo-S, Ca-Mg, Cu-Mn and Cu-Mg concentrations in response to different Fe supplies were positively correlated in at least four of the five organs analyzed.

**Conclusions:**

Mineral content loss analysis indicated that mineral remobilization from vegetative organs can occur in rice plants; however, for seeds to acquire minerals, vegetative remobilization is not absolutely required. Also, mineral remobilization from vegetative tissues in rice was greatly dependent of plant Fe nutrition. Remobilization was observed for several minerals from flag leaves and stems/sheaths, but the amounts were generally far below the total mineral accretion observed in panicles, suggesting that continued uptake and translocation of minerals from the roots during seed fill are probably more important than mineral remobilization.

**Electronic supplementary material:**

The online version of this article (doi:10.1186/1939-8433-5-27) contains supplementary material, which is available to authorized users.

## Background

Plants are the primary source of nutrients for human nutrition on a global basis. Staple seed crops, such as rice, supply the majority of daily dietary nutrients for billions of people. However, rice has a low density of mineral nutrients, and for those whose diets are high in staple foods, micronutrient malnutrition is widespread (Grusak and DellaPenna^1999^). Biofortification, which consists of the use of plant breeding and/or transgenic approaches to develop new cultivars with the potential to increase the nutrient concentration of edible portions of crop plants (White and Broadley^2005^), has emerged as one possible solution to alleviate malnutrition. Despite the increasing number of studies about the physiology and regulation of uptake of several minerals from the rhizosphere, the lack of knowledge about how minerals are moved into or out of vascular tissues, translocated to vegetative tissues and loaded into seeds is one of the barriers to seed biofortification (Colangelo and Guerinot^2006^).

Initial seed biofortification efforts for Fe and Zn in rice have focused on increasing the iron storage protein ferritin (Goto et al.^1999^; Vasconcelos et al.^2003^) or root ferric reductase activity (Vasconcelos et al.^2004^). The transgenic plants showed overaccumulation of minerals in leaves, but only a small increase in seeds. Grain Zn and Fe concentrations were increased in barley (*Hordeum vulgare*) expressing the Zn transporter *ZIP1* from *Arabidopsis thaliana* (Ramesh et al.^2004^), and were decreased in wheat (*Triticum aestivum*) expressing RNAi constructs that lowered *NAM* family gene expression (Uauy et al.^2006^). Vasconcelos et al. (2006) constitutively expressed the root ferric reductase *FRO2* from *A. thaliana* in soybean and observed that, in certain hydroponic growth conditions, the transgenic plants showed a threefold increase in leaf Fe concentration, but only a 10% increase in seed Fe. Similar results were found with the *brz* mutant of *Pisum sativum*, which overaccumulates Fe in leaves but has normal seed Fe concentrations (Grusak^1994^). Analysis of mineral overaccumulation mutants indicates that translocation of minerals to seeds is tightly regulated, and that simply increasing net mineral uptake into the plant will probably not result in seeds with higher mineral concentrations. Additional regulatory mechanisms or transport capabilities must be manipulated to improve remobilization and pass through of minerals from vegetative organs into seeds. Recently, over-expression of barley genes related to phytosiderophore synthesis resulted in enhanced Fe and Zn concentrations in rice unpolished and polished seeds (Masuda et al.^2008^; Masuda et al.^2009^). Enhanced expression of the three genes from the rice nicotianamine synthase family (*OsNAS* genes) also facilitated increases in Fe and Zn concentrations of rice grains (Lee et al.^2009^; Zheng et al.^2010^; Johnson et al.^2011^; Lee et al.^2011^). All of these results suggest that the most successful breeding or transgenic approaches will likely need to target multiple genes simultaneously (Waters and Grusak^2008^, Sperotto et al.^2012^).

It is accepted that minerals may be remobilized from vegetative sources (Hocking and Pate^1977^; Himelblau and Amasino^2001^), although a major portion of minerals in seeds are likely supplied through continuous uptake and translocation during reproductive growth to developing seeds. In wheat, Zn (Hocking^1994^) and Fe (Garnett and Graham^2005^) remobilization from leaves was observed. According to Jiang et al. (2007b), when ^65^Zn is applied to rice leaves (either the flag leaf or the lowest senescent leaf), 45-50% is transported out of the treated leaf. From that Zn, more than 90% is translocated to other vegetative organs; little is partitioned to the panicle parts and even less to the grains. These results suggest that, in rice plants grown under sufficient or surplus Zn supply, most of the Zn accumulated in the grain originates from uptake by roots after flowering and not from Zn remobilization from leaves. In another study, Zn was applied to rice plants in various amounts and at various stages of plant development. Analysis of the total plant Zn and Zn content in individual organs at flowering and at maturity indicated that the presence of Zn in grains could be fully attributed to additional Zn uptake after flowering, except when application rates were very low (Jiang et al.^2008^). Under the latter conditions, roots, stems and sheaths contributed mostly to Zn allocation. None of the rice Zn-application treatments showed that the main portion of Zn loaded in grain was remobilized from leaves (Jiang et al.^2008^; Stomph et al.^2009^). Contrastingly, Wu et al. (2010) showed that large amounts of the Zn deposited in the rice grains at maturity had been retranslocated from other plant parts and not been transported directly after uptake to the grains in the grain-filling stage. Recently, Yoneyama et al. (2010) reported that Zn in the rice grains and partly in the husks may be actively supplied via the phloem after mobilization from the blades of the flag and upper leaves and also by xylem-to-phloem transfer in the nodes. Fe stored in the flag and upper leaves may be transported to the grains via the phloem. Fang et al. (2008) showed that foliar application of Zn and Se can influence the Zn, Se, and Fe content of rice grains; however, it was difficult to improve the Fe nutrition of rice grain by Fe spray, probably due to its limited mobility in the phloem. Waters and Grusak (2008) suggested that, in *Arabidopsis*, continuous uptake and translocation of minerals to source tissues during seed fill are as important, if not more important, than remobilization of previously stored minerals.

Uncovering the genetic architecture underlying mineral ion homeostasis in plants is a critical first step towards understanding the biochemical and physiological processes that regulate a plant’s elemental composition. In this paper, we assess growth dynamics of the whole plant (panicles, non-flag leaves, flag leaves, stems/sheaths and roots) over the reproductive development of *Oryza sativa*. We also describe the concentrations and contents of ten mineral nutrients (Fe, Zn, Cu, Mn, Mo, Ni, Ca, Mg, K and S) in these organs over time. Additionally, we studied the net loss of mineral contents to investigate the potential of vegetative tissues as sources of stored minerals to be remobilized to panicles. To address the question of whether different Fe supplies could influence mineral partitioning and movement of minerals to panicles, we compared these parameters in plants treated with low, normal and high Fe concentrations.

## Results

**Figure 1:**
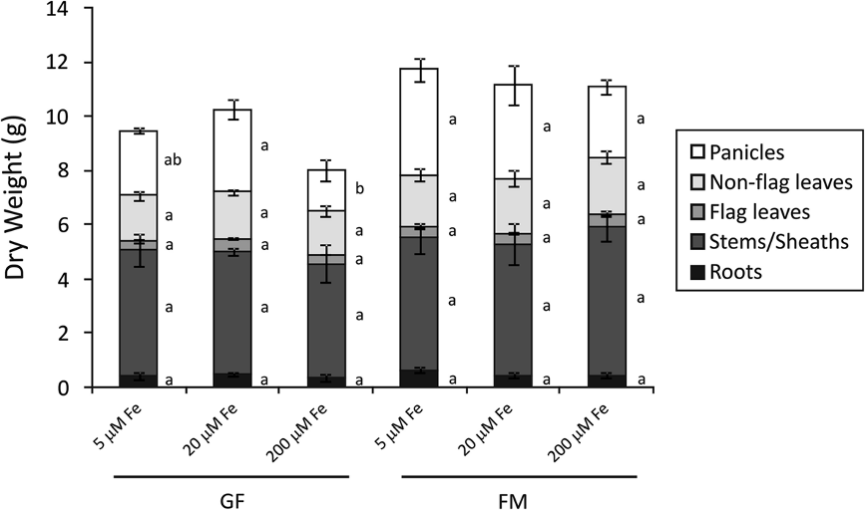
**Dry weight of rice organs.** Dry weight of panicles, non-flag leaves, flag leaves, stems/sheaths and roots collected during grain filling (GF) and full maturity (FM) stages of rice plants supplied with 5, 20 or 200 μM of Fe(III)-HEDTA from panicle exertion (PE) onwards. Values are the averages of at least three samples ± SE. Different letters indicate that the means (between different Fe supplies) are different by the Tukey HSD test (*P* ≤ 0.05). Error bars may be too small to be visible in the figure.

To test if different Fe supplies would influence mineral accumulation in rice organs, we cultivated Kitaake plants with 5, 20 or 200 μM of Fe(III)-HEDTA from the panicle exertion (PE) until the full maturity (FM) stage. Different Fe supplies caused slight growth changes in panicles collected at the grain filling (GF) stage, while no changes were seen at the full maturity stage (Figure1). High Fe concentration (200 μM) caused a decrease of about 50% in panicle dry weight compared to the control concentration (20 μM). However, no impact on dry weight was seen in other organs, possibly because the high Fe treatment used in this work did not lead to enough Fe accumulation to reach toxic levels (300 μg g^-1^ DW – Sahrawat^2004^) in all aerial plant organs (Additional file1). A panicle weight reduction of about 22% (not statistically significant) was seen when plants were cultivated with limited Fe supply (5 μM), relative to 20 μM Fe controls. Therefore, the different Fe supplies used in this work during reproductive development did not result in different dry weight in most organs. Changes in mineral contents (Additional file2) were mainly dependent on changes in mineral concentrations.

Most of the changes in mineral concentrations resulting from different Fe supplies were found during full maturity stage. Panicle (along with flag leaf) was the organ with the least variation in mineral concentrations caused by different Fe supplies (Additional file1, Additional file3 and Additional file4). At GF stage, only Fe (Additional file1a), Ni (Additional file3k) and Mg (Additional file4f) concentrations showed differences due to the Fe supply. However, the decrease in panicle dry weight detected in plants cultivated with high Fe concentration during GF stage (< 50%; Figure1) resulted in decreased content in most of the minerals analyzed, except Fe, Mo and K (Additional file2). At FM stage, none of the minerals analyzed showed difference in the content between control (20 μM) and high Fe (200 μM) condition (Additional file2).

In non-flag leaves, no obvious mineral concentration dynamic could be detected. High Fe supply was responsible for higher Fe concentration (Additional file1b) and for lower Cu (Additional file1L), Mo (Additional file3g), Ca (Additional file4b) and S (Additional file4q) concentrations. Low Fe supply was responsible for higher Mn (Additional file3b) and Ni (Additional file3L) concentrations during GF and FM; Ca (Additional file4b) and Mg (Additional file4g) during FM and K (Additional file4L) during GF stage.

In flag leaves, most of the mineral concentrations (including Fe and Zn) were not affected by different Fe supplies. During GF stage, only Mo (Additional file3h), Mg (Additional file4h) and S (Additional file4r) concentrations were reduced by low and/or high Fe concentrations. During FM stage, along with Mo, Mg and S, the concentrations of Cu (Additional file1m), Mn (Additional file3c) and Ca (Additional file4c) also presented variations according to the Fe supply.

In stems/sheaths, most of the modifications in mineral concentrations found in plants collected at the GF stage were under high Fe supply. Only Fe (Additional file1d), Cu (Additional file1n) and Ni (Additional file3n) maintained similar concentrations independent of the Fe supply. During FM stage, Fe was the only mineral whose concentration increased at 200 μM Fe (Additional file1d). All the other minerals reached higher mineral concentrations at 5 μM Fe.

In roots, all the minerals analyzed, except K (Additional file4o) and S (Additional file4t), presented different concentrations when plants were cultivated with different Fe supplies. As expected, root Fe concentration was higher under 200 μM Fe (2.2X in GF and 4.2X in FM; Additional file1e). The opposite was found for Zn (Additional file1j), Cu (Additional file1o), Mo (Additional file3j) and Ni (Additional file3o), with higher concentrations under lower Fe supply. K (Additional file4o) and S (Additional file4t) concentrations were not affected by different Fe supplies.

**Figure 2:**
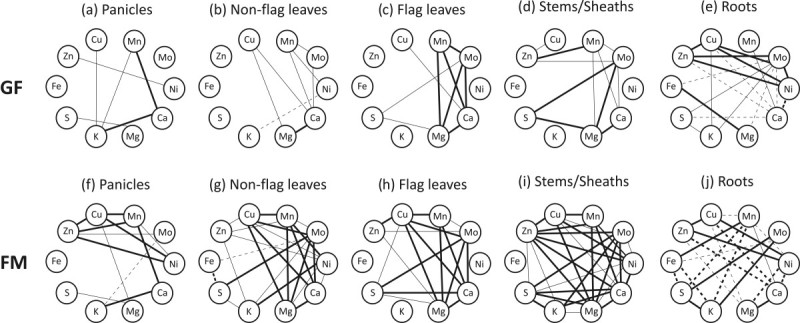
**Mineral correlation analyses.** Pearson’s correlation analysis of ten mineral concentrations in panicles (**a** and **f**), non-flag leaves (**b** and **g**), flag leaves (**c** and **h**), stems/sheaths (**d** and **i**) and roots (**e** and **j**) of rice plants cultivated with 5, 20 and 200 μM of Fe(III)-HEDTA during grain filling (GF) and full maturity (FM) stages. Solid lines represent a significant positive correlation and dashed lines represent a significant negative correlation. Thinner lines indicate significance at the 0.05 level and thicker lines indicate significance at the 0.01 level.

Pearson’s correlation analysis was performed in order to find relationships among the ten mineral concentrations in response to the different Fe supplies. As seen in Figure2, most of the correlations were found during full maturity (FM) stage, after a longer period under different Fe supplies. Negative correlations were found mainly in roots. During grain filling (GF) stage, the only pair of minerals positively correlated in every tested organ was Mn-Ca. These were also positively correlated during FM stage in all tested organs, along with Zn-Cu. Positive correlations at FM between the pairs Zn-Ni and Cu-Ni were found in four of the five tested organs, except in flag leaves. Similar dynamic was found for Mo-S and Ca-Mg, which were not correlated only in panicles. Cu-Mn and Cu-Mg were also positively correlated in four of the five tested organs, except in roots, showing a negative correlation in this organ (Figure2).

**Figure 3:**
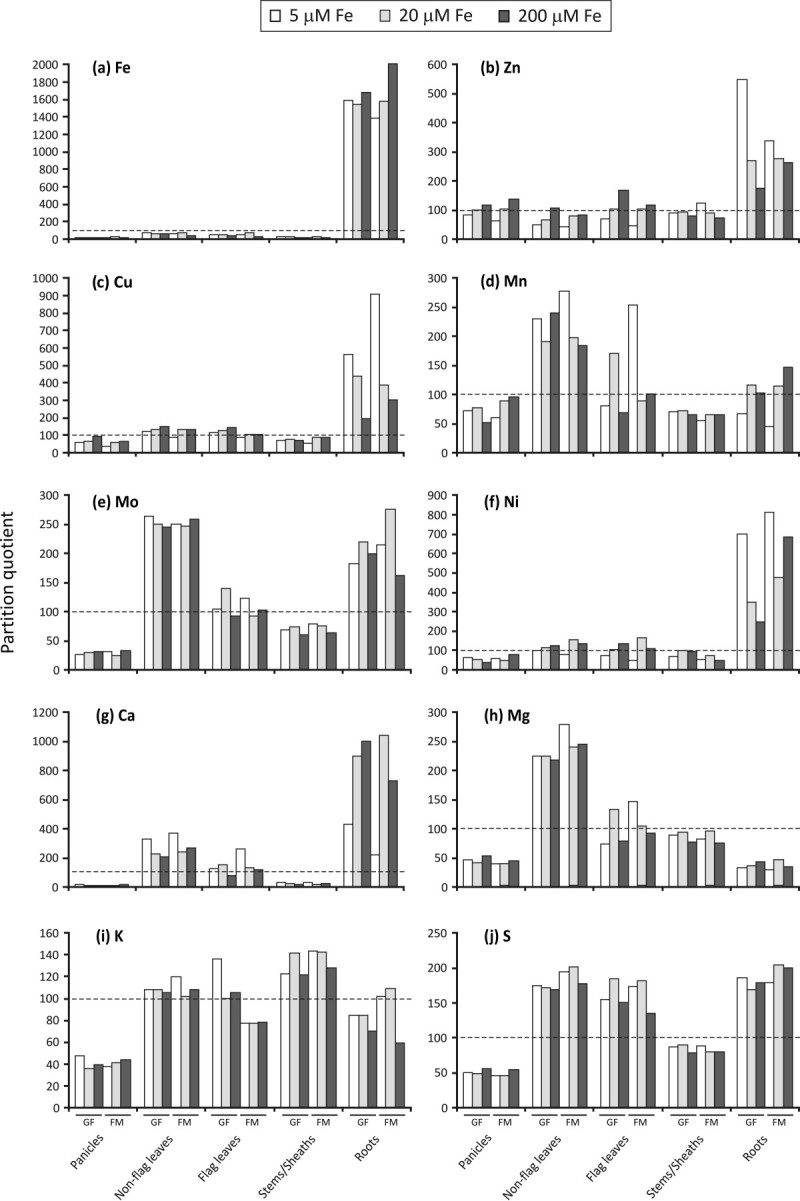
**Partition quotients (PQ) analyses.** Partition quotients of mineral elements in panicles, non-flag leaves, flag leaves, stems/sheaths and roots during grain filling (GF) and full maturity (FM) stages of rice plants supplied with 5, 20 or 200 μM of Fe(III)-HEDTA from panicle exertion onwards. Dashed horizontal line represents PQ of 100 (the percentage contribution of the organ to the plant’s dry weight is the same as the percentage contribution to the plant’s total content of the mineral being evaluated).

Differences in plant size were responsible for the high standard error in mineral contents (Additional file2). Therefore, partition quotient (PQ) values were calculated to allow comparison of the dynamics of partitioning of minerals between the reproductive development of rice plants cultivated with different Fe supplies (Figure3), since this approach is able to check if one mineral content is higher in one specific organ, regardless of differences in plant size. In general, non-flag leaves, flag leaves and roots were the organs which showed the higher mineral PQ variation according to Fe supply, whereas stems/sheaths was the organ with lower variation (Figure3). For Fe, PQ values above 100 were found only in roots (Figure3a), with higher values in plants cultivated with 200 μM Fe. In all the other organs, high Fe supply was responsible for the lowest Fe PQ value, especially during FM (Additional file5). Also, in this stage, control Fe supply (20 μM Fe) resulted in higher PQ values (Additional file5). For Zn, low Fe supply resulted in the lowest Zn PQ values in panicles, non-flag leaves and flag leaves, and high Fe supply resulted in the highest Zn PQ values (Figure3b). In stems/sheaths (during FM) and roots (during both stages), the opposite was found, with higher Zn PQ values under low Fe supply (Figure3b). Similar pattern was found for Cu (Figure3c) and Ni (Figure3f) PQ in roots. For Mn, no obvious pattern could be identified, except the higher PQ values in non-flag and flag leaves under Fe deprivation and in panicles and roots under Fe excess, both during FM (Figure3d). Mo (Figure3e) and S (Figure3j) were the minerals with the most stable PQ values. For Ca (Figure3g) and Mg (Figure3h) high PQ values were obtained in non-flag and flag leaves during FM stage under Fe starvation. Low Fe supply also resulted in extremely reduced Ca PQ values in roots (Figure3g), in relation to values seen in the other treatments.

**Figure 4:**
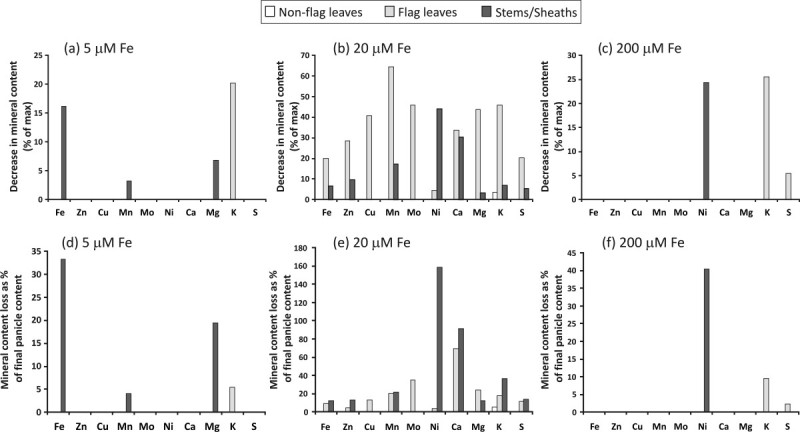
**Mineral remobilization analyses.** Percentage decrease in non-flag leaves, flag leaves and stems/sheaths mineral contents from grain filling to full maturity stages (**a**) and potential contribution of remobilized mineral to panicle mineral contents at the final time point (**b**).

To investigate the potential of vegetative tissues as a source of stored minerals to be remobilized to panicles, we estimated the net loss of mineral content from non-flag leaves, flag leaves and stems/sheaths, by subtracting final mineral content from the prior time point which had the highest mineral content. As seen in Figure4, net loss of mineral content could be discerned under different Fe supplies. Under control Fe concentration (20 μM), all the minerals showed content loss from GF to FM stage (Figure4b), mostly from flag leaves but also from stems/sheaths. However, as the flag leaves’ mineral content is much lower than the stems/sheaths’ content, the maximum possible contribution to panicle mineral content, assuming that the total net loss of each mineral was translocated to panicles before the final collection point, is in general higher from stems/sheaths than from flag leaves. Different Fe supplies influenced the content loss of most of the minerals. Under limited Fe condition (5 μM), only Fe, Mn and Mg showed a net remobilization from stems/sheaths; and K from flag leaves. Under high Fe condition (200 μM), only K and S showed a net remobilization from flag leaves; and Ni from stems/sheaths. Surprisingly, no remobilization was found from non-flag leaves, except a minimum K remobilization under 20 μM Fe, which could have contributed at most only with 5% of total panicle K content (Figure4e). For most of the minerals analyzed, except Ni and Ca, the maximum possible contribution of remobilization from flag leaves and stems/sheaths to the final panicle’s mineral content is less than 40%, being less than 20% for Fe, Zn, Cu, Mn and S. Ni and Ca remobilization from flag leaves and mostly from stems/sheaths could be responsible for more than 100% of the final panicle content (Figure4e), showing that remobilization does not necessarily correspond to mineral translocation to the panicles.

To determine whether different Fe supplies could result in different mineral export to developing seeds, we submitted rice plants to the same Fe conditions as the previous experiment (although 0 μM Fe was included in this analysis). In general, the dynamics of mineral concentrations of seeds (Additional file6) were similar to those of panicles; that is, higher Fe and lower Zn and Mo concentrations under 200 μM Fe. Fe, Cu, Mn, Mg and S showed the lowest and Zn, Mo and Ni showed the highest concentrations under Fe deprivation (Additional file6).

## Discussion

Plants are commonly faced with fluctuations in nutrient supply to the roots. To cope with these fluctuations, plants invoke a range of mechanisms, which include different uptake rates, changes in root morphology and physiology or storage and remobilization of mineral nutrients (Marschner^1995^). In this study, we characterized organ-specific changes in dry matter and mineral (Fe, Zn, Cu, Mn, Mo, Ni, Ca, Mg, K and S) content to monitor the net flow of minerals into and through the rice plant over the reproductive development and under different Fe supplies. Any increase in mineral content in one organ must have resulted from uptake and translocation from the soil, or from remobilization from one organ to another. By harvesting all the organs and tracking the mineral partitioning in these organs over time, remobilization can be estimated for each mineral. In this work we used the same remobilization definition used by Waters and Grusak (2008), which is defined as the net loss of stored or recycled mineral content from one organ over time, with the mineral loss representing movement into another organ.

### Effects of low and high Fe supplies on mineral dynamics

As shown in Additional file2 and Figure3, low Fe supply (5 μM) allows higher accumulation of Zn, Cu and Ni in roots; Mn, Ca, Mg and K in leaves and Zn in stems/sheaths. On the other hand, low Fe supply allows a smaller accumulation of Fe, Mn and Ca in roots and Zn and Ni in leaves. Enhancement of Zn uptake under low Fe supply, either as a divalent cation or complexed to phytosiderophores (Zhang et al.^1998^), could explain its higher accumulation in roots. Several Fe transporters are able to transport also Zn and Mn (Korshunova et al.^1999^; Eckhardt et al.^2001^; Gross et al.^2003^; López-Millán et al.^2004^). Induction of Fe transporters under low Fe concentration could result in increased Zn uptake and translocation to stems/sheaths and increased Mn uptake and subsequent translocation to leaves, since Mn moves easily from root to shoot in the xylem-sap transpiration stream (Ramani and Kannan^1987^). Possibly, uptake of Mn and Cu may increase in rice plants under low Fe supply, because the presence of phytosiderophores in the rhizosphere may increase the availability of these ions both in the rhizosphere itself and in the apoplast (Zhang et al.^1991^). The complex Cu-phytosiderophore can be transported into root cells by Fe-phytosiderophore transporters, although with lower affinity (Briat et al.^1995^). Fertilizing a calcareous soil with an Fe-deficient solution increased Cu accumulation by roots and shoots in two wheat cultivars (Chaignon et al.^2002^). It has been suggested that Cu, Zn and Ni share a common uptake system (Kochian^1991^), which could explain the higher accumulation of Ni in roots under low Fe supply. In fact, Eckhardt et al. (2001) proposed that the Fe transporters IRT1 and IRT2 from tomato could also transport Ni^2+^. Differently from our results, Silveira et al. (2007) detected increased Ca and K concentrations under Fe-deficiency in roots of rice, instead of in leaves. Possible explanations for such difference in Ca dynamic under low Fe supply are that we have used a different rice cultivar, a completely different technique for mineral detection (ICP-OES versus PIXE), different levels of Fe supply (5 μM Fe versus Fe deprivation) and we analyzed rice organs through the course of reproductive development instead of during vegetative development.

In rice, Fe toxicity seems to occur above 500 μg Fe g^-1^ leaf dry weight (Audebert^2006^) and leads to a drastic reduction of root growth (Becker and Asch 2005). As shown in Figure1 and Additional file1, our plants cultivated with high Fe supply did not present reduction of root growth and did not reach toxic levels. However, the treatment resulted in decreased dry weight of panicles collected during grain filling (Figure1). In fact, depending on the site and the cultivars used, reported critical concentrations can range from 20 to 2,500 μg g^–1^, indicating that factors other than Fe concentration influence the occurrence of Fe toxicity symptoms (Becker and Asch^2005^). In this way, the fluctuations in mineral accumulations found in the present work do not reflect the changes promoted by Fe toxicity. High Fe supply (200 μM) promoted a higher accumulation of Fe in roots and Zn in leaves. On the other hand, it promoted a smaller accumulation of Fe in leaves and stems/sheaths and Zn, Cu and K in roots (Additional file2, Figure3 and Additional file5). As expected, high Fe supply resulted in higher Fe accumulation in roots. However, root Fe measurements could be over-estimated. The oxidation ability of the rice root precipitates inactive Fe at the root apoplast and root-environment interface. The sampling technique used did not separate the existing oxidized Fe at the root surface. Thus, the root Fe content measured comprised the root Fe uptake and the oxidized Fe at the root surface. Most of this Fe seems to not be translocated to shoots, since smaller Fe PQ values were detected in leaves and stems/sheaths (Figure3), even with the increased Fe content in non-flag leaves (Additional file2). Under Fe-excess (500 mg L^-1^), Silveira et al. (2007) reported that rice roots and also shoots accumulate higher Fe concentration. In that work, higher concentrations of Fe were detected in I409 plants, more susceptible to Fe toxicity. Shoot concentrations detected were up to 2.5X higher than in E108 plants (tolerant to Fe toxicity) after 10 days of treatment. The authors suggested, therefore, that E108 plants were more resistant to excess Fe due to the possible induction of avoidance mechanisms, allowing the plant to decrease Fe translocation to shoots. This avoidance mechanism was recently reviewed by Sperotto et al. (2010). In our work, Kitaake plants seem to use the avoidance mechanism, but additional studies are needed to confirm the hypothesis that Kitaake is an Fe-excess-tolerant rice cultivar.

In accordance with our results, Silveira et al. (2007) also detected higher Zn concentration in shoots of plants under Fe-excess. Also in accordance to our results, smaller K (Neue et al.^1998^; Ramirez et al.^2002^; Mehraban et al.^2008^) and Cu (Shao et al.^2007^; Silveira et al.^2007^) absorption rates under Fe-excess have already been reported in rice. It seems that, under Fe-excess, the relatively high ferrous iron concentration in the soil solution and uptake by the plant may result in K deficiency (Audebert^2006^). On the other hand, it appears that proper K supply may increase Fe exclusion from roots and reduce translocation of Fe to aerial plant organs, especially to upper leaves (Sahrawat^2004^).

Significant correlations were found among the ten mineral element concentrations in rice organs of plants facing different Fe supplies. In particular, Mn-Ca and Zn-Cu were positively correlated in all analyzed organs. Zn-Ni, Cu-Ni, Mo-S, Ca-Mg, Cu-Mn and Cu-Mg were positively correlated in four of the five organs (Figure2). Most of the available data about mineral correlation in plants were obtained in grains. As far as we know, this is the first work which tries to identify similar patterns of mineral fluctuations in different plant organs due to different Fe supplies. In this way, significant positive correlations between minerals suggest that different Fe supplies promote similar changes in their concentrations. Fe concentration was positively correlated with only two minerals (Mn and Mg) in roots (Figure2e and j). It is already known that Fe transporters can also transport Mn (Korshunova et al.^1999^; Eckhardt et al.^2001^; Gross et al.^2003^; López-Millán et al.^2004^) and high positive correlation between Fe and Mn was found in rice grains, along with Mn-Ca, Zn-Cu, Ca-Mg, and Cu-Mn (Jiang et al.^2007^a), in accordance with our findings. However, no Fe-Mn correlations were found in other rice organs. One possible explanation is that Mn moves easily from the root to the shoot in the xylem-sap transpirational stream (Ramani and Kannan^1987^), and the mobility of Fe is more regulated (Marschner 1995). The physiological significance of the correlation between Fe-Mg is unknown, but Jiang et al. (2007a) have already detected such relation in milled rice. These authors suggest that the high number of positive correlations in rice is probably due to the interaction between ions whose chemical properties are sufficiently similar, and such similarity allows the competition for site of absorption, transport and function in plant tissues. One example was shown by Kupper and Kupper (1998), which reported that the heavy metals (Hg, Cu, Cd, Ni, and Pb) might substitute Mg, the central atom of chlorophyll. This could help to explain the positive correlations we found between Cu and Mg in all the organs, except in roots (Figure2). Zeng et al. (2005) also found that Mn is positively associated with Ca in rice grains and Majumder et al. (1990) reported that uptake of Mn and Ca are positively correlated in rice cultivated in P-deficient soil, along with uptake of Ca and Mg.

Various molecules are known to bind minerals in rice organs and could explain some of the correlations detected, such as Ca-K in panicles, because both minerals bind to phytic acid in the aleurone layer of rice grain (Lin et al.^2005^). Similarly, many of the micronutrient correlations found in various organs could be attributed to nicotianamine or deoxymugineic acid, as these are known chelators of several metal cations in plant tissues (Curie et al.^2009^). However, further studies are needed to confirm the possibility of the same chelating molecules being involved in the mineral correlations detected.

### Mineral remobilization versus continued supply from roots

The dynamics of mineral concentrations in seeds of plants cultivated with different Fe supplies showed that higher Fe supply results in higher Fe concentration in the seeds (Additional file6), implying that the uptake and transport systems were operating below capacity when plants were grown with lower Fe concentrations. As previously shown by Fang et al. (2008), it is difficult to improve the Fe nutrition of rice grain, even by Fe spray, possibly due to its limited mobility in the phloem. Probably, enhanced root uptake combined with leaf/stem/sheath efflux transport can be an effective way to increase the Fe concentration in rice grains.

Remobilization of reserves to supply rice seeds with minerals has been emphasized in previous studies (Jiang et al.^2007^b; Fang et al.^2008^; Jiang et al.^2008^; Wu et al.^2010^; Yoneyama et al.^2010^), but the contribution of stored minerals to total seed mineral content is unclear. According to Waters and Grusak (2008), it is expected that a minimal amount of each mineral is incorporated into structural or protein molecules and thus unavailable for mobilization, and that source tissues would have to accumulate minerals in excess of this minimal amount to allow mobilization to growing tissues such as seeds. This was the case for S in soybean leaves (Sunarpi and Anderson^1996^), where a soluble S pool was available for remobilization and an insoluble S pool could not be mobilized. The size of the soluble pool was dependent on S nutrition. In the present study, plants fertilized with a high Fe concentration (200 μM) showed only K and S remobilization from flag leaves and Ni from stems/sheaths (Figure4). In this case, vegetative tissues should have been able to store Fe quantities above the structural minimum, which would have provided excess Fe for remobilization. However, with the abundant Fe supply at the root level, continued uptake during seed fill may have reduced or precluded the need for remobilization to serve as a source of Fe for seeds. On the other hand, plants fertilized with low Fe concentration (5 μM) showed the highest net Fe remobilization (Figure4), probably due to reduced uptake during seed fill. Under limited Fe supply, stems/sheaths are the major Fe source for remobilization. A similar pattern was observed in other studies when Zn supply was very low. Under this condition, stems and sheaths contributed most to Zn allocation (Jiang et al.^2008^). All the minerals analyzed were subject to remobilization, mostly from flag leaves, but also from stems/sheaths (Figure4), when plants were fertilized with the control Fe concentration (20 μM). These results suggest that mineral remobilization from vegetative tissues can occur in rice plants; however, for seeds to acquire minerals this remobilization is not absolutely required. Remobilization results found for *Arabidopsis* plants byWaters and Grusak (2008) were not exactly consistent between experiments; differences were found in the minerals that were remobilized and the amounts remobilized. Crafts-Brandner (1992) also found inconsistent results in leaves of soybean, which remobilized P in one experiment, while in a second experiment no remobilization occurred, yet seeds of both experiments had comparable seed P concentrations. As shown by our results, mineral remobilization from vegetative tissues in rice is modified by plant Fe nutrition, because different Fe supplies alter the remobilization of several minerals. Also, a major proportion of mineral content in panicles probably comes from non-storage sources, that is, continued root uptake and translocation during the seed fill period. We did observe mineral remobilization mostly from flag leaves but also from stems/sheaths with 20 μM Fe, and at least a portion of these minerals was likely incorporated into panicles and seeds. In the unlikely event that 100% of the content of each mineral lost from vegetative tissues went to panicles, this could account for, at most, 20% of panicle Fe, Zn, Cu, Mn, Mg, K and S. However, at least for Zn, it is already known that most of the mineral transported out of the leaves (either the flag leaf or the lowest senescent leaf) is translocated to other vegetative organs instead of being partitioned to the panicles, and even less to the grains (Jiang et al.^2007^b). We can also discard the possibility of 100% Ni or Ca remobilization to panicles, since it would be responsible for more than 100% of the final panicle content (Figure4). However, if remobilized, a significant part of this Ni pool would be expected to be transported to the seeds, since it is already known that Ni rapidly re-translocates from leaves to young tissues in the phloem, particularly during reproductive growth. Indeed, up to 70% of Ni in the shoots was transported to the seeds of soybean (Tiffin 1971). In *Arabidopsis*, continuous uptake and translocation of minerals to source tissues during seed fill are as important, if not more important, than remobilization of previously stored minerals (Waters and Grusak 2008). As shown by our results, the flag leaf is the organ with highest percentage of mineral content loss. However, considering that flag leaves have extremely low mineral content, the maximum possible contribution to panicle mineral content would be from stems/sheaths, rather than from flag leaves.

## Conclusion

In summary, this study suggests that mineral accumulation throughout the reproductive development of rice organs can be affected by different Fe supplies. With respect to the translocation of minerals to panicles, remobilization from vegetative organs can occur in rice plants (and non-optimal Fe supplies can affect mineral remobilization), but apparently, for seeds to acquire minerals, vegetative remobilization is not absolutely required. In this way, continued uptake and translocation of minerals during seed fill are probably more important than remobilization of previously stored shoot minerals. Thus, in addition to targeting source tissues for increased mineral remobilization, researchers should also target root uptake and leaf/stem/sheath efflux transporters to increase mineral accumulation in the panicles and consequently in the rice seeds.

## Methods

### Plant materials and growth conditions

Rice (*Oryza sativa* L.) seeds from the fast-growing cultivar Kitaake were germinated in Petri dishes with filter paper for 8 d before being transferred to hydroponic solution. Plants were grown in a controlled environment chamber with 16-h, 20°C day and 8-h, 15°C night at the USDA-ARS Children’s Nutrition Research Center, Houston, TX. Relative humidity was maintained at 50% and photon flux density during the day was 350 μmol m^–2^ s^–1^, supplied by a mixture of incandescent bulbs and fluorescent lamps. The standard solution for hydroponically grown plants contained 1 mM Ca(NO_3_)_2_, 3 mM KNO_3_, 0.5 mM MgSO_4_, 0.75 mM K_2_SO_4_, 0.5 mM KH_2_PO_4_, 25 μM CaCl_2_, 25 μM MnSO_4_, 0.5 μM ZnSO_4_, 0.5 μM CuSO_4_, 0.5 μM H_2_MoO_4_, 0.1 μM NiSO_4_, 0.1 mM K_2_SiO_3_, and 20 μM Fe(III)-HEDTA (N-hydroxyethyl-ethylenediaminetriacetic acid). All nutrients were buffered with 2 mM MES (2,4-morpholino-ethane sulfonic acid), pH 5.5 and growth solutions were replaced every 3 days. For treatments involving different Fe concentrations, Fe(III)-HEDTA concentration was adjusted to 5, 20 or 200 μM. Rice organs (panicles, non-flag leaves, flag leaves, stems/sheaths and roots) were collected at panicle exertion, grain filling and full maturity (R3, R5 and R9 stages, respectively, according to Counce et al.^2000^). De-husked unpolished seeds were analyzed at full maturity.

### Elemental analysis by ICP

All tissues were harvested and dried in a 60°C oven for 48 h. Dried tissues were predigested overnight in borosilicate glass tubes with 4 ml of redistilled 98.8% HNO_3_. One milliliter of concentrated trace metal grade HClO_4_ was added to the predigested tissues and heated at 100°C for 1 h, 150°C for 1 h, 180°C for 1 h and then at 210°C to dryness (1–2 h). Digestions were performed using a heating block (Model 1016, Tecator, Hoganas, Sweden) with an exhaust-collecting manifold. Digests were resuspended in 15 ml of redistilled 2% HNO_3_. Concentrations of Fe, Zn, Cu, Mn, Mo, Ni, Ca, Mg, K and S were determined by inductively coupled plasma-optical emission spectroscopy (CIROS ICP Model FCE12; Spectro, Kleve, Germany). Tomato leaves and rice flour standards (SRM 1573A and 1568A, respectively; National Institute of Standards and Technology, Gaithersburg, MD) were digested and analyzed along with the rice samples to ensure accuracy of the instrument calibration (Narayanan et al.^2007^). Mineral content was determined by multiplying each sample’s concentration by dry weight. For estimation of net mineral content loss from non-flag leaves, flag leaves and stems/sheaths, the final mineral content of each organ was subtracted from the prior time point which had the highest mineral content. For minerals that had a decrease in content, the net loss was compared with final total panicle mineral content to determine the contribution of remobilized minerals to panicle mineral content.

### Partition quotient calculation

To evaluate the partitioning of minerals within a rice plant during its reproductive development and under different Fe supplies, changes in each tissue’s content were normalized to changes in each tissue’s weight, relative to the whole plant. The DW of each organ was calculated as a percentage of total plant weight at each time point (or at each Fe supply), and mineral content of each organ was calculated as a percentage of total plant mineral content at each time point (or at each Fe supply). Using these values, the normalized partitioning of each mineral within the plant was calculated by dividing each organ’s percentage mineral content by its percentage DW, and multiplying by 100, which we refer to as the partition quotient (PQ), as described by Waters and Grusak (2008).

### Statistical analyses

When appropriate, data were subjected to analyses of variance (ANOVA) and means were compared by the Tukey HSD (Honestly Significant Differences) (*P* ≤ 0.05). The Levene’s test (for homogeneity of variance) was used prior to ANOVA. Data with unequal variances were subjected to Welch analysis and means were compared by the Dunnett-C test. Pearson’s correlation analyses were carried out using two significance levels (*P* ≤ 0.05 and 0.01). All the statistical analyzes were performed using the SPSS Base 19.0 for Windows (SPSS Inc., USA).

## Electronic supplementary material

Additional file 1: **Fe, Zn and Cu concentrations.** Fe, Zn and Cu concentrations in panicles, non-flag leaves, flag leaves, stems/sheaths and roots collected during grain filling (GF) and full maturity (FM) stages of rice plants cultivated with 5, 20 or 200 μM of Fe(III)-HEDTA. Values are the averages of at least three samples ± SE. Different letters indicate that the means (between different Fe supplies) are different by the Tukey HSD test (*P* ≤ 0.05). Error bars may be too small to be visible in the figure. (JPEG 3 MB)

Additional file 2: **Mineral contents in rice organs.** Contents of Fe, Zn, Cu, Mn, Mo, Ni, Ca, Mg, K and S in panicles, non-flag leaves, flag leaves, stems/sheaths and roots collected from rice plants supplied with different Fe concentrations at two reproductive growth stages. (DOCX 23 KB)

Additional file 3: **Mn, Mo and Ni concentrations.** Mn, Mo and Ni concentrations in panicles, non-flag leaves, flag leaves, stems/sheaths and roots collected during grain filling (GF) and full maturity (FM) stages of rice plants cultivated with 5, 20 or 200 μM of Fe(III)-HEDTA. Values are the averages of at least three samples ± SE. Different letters indicate that the means (between different Fe supplies) are different by the Tukey HSD test (*P* ≤ 0.05). Error bars may be too small to be visible in the figure. (JPEG 3 MB)

Additional file 4: **Ca, Mg, K and S concentrations.** Ca, Mg, K and S concentrations in panicles, non-flag leaves, flag leaves, stems/sheaths and roots collected during grain filling (GF) and full maturity (FM) stages of rice plants cultivated with 5, 20 or 200 μM of Fe(III)-HEDTA. Values are the averages of at least three samples ± SE. Different letters indicate that the means (between different Fe supplies) are different by the Tukey HSD test (*P* ≤ 0.05). Error bars may be too small to be visible in the figure. (JPEG 4 MB)

Additional file 5: **Iron partition quotient (PQ) in rice organs.** Iron partition quotient in panicles, non-flag leaves, flag leaves and stems/sheaths during grain filling (GF) and full maturity (FM) stages of rice plants cultivated with 5, 20 or 200 μM of Fe(III)-HEDTA. (JPEG 388 kb) (JPEG 388 KB)

Additional file 6: **Mineral concentrations in rice seeds.** Fe, Zn, Cu, Mn, Mo, Ni, Ca, Mg, K and S concentrations in de-husked unpolished seeds collected during full maturity stage of rice plants cultivated with 0, 5, 20 or 200 μM of Fe(III)-HEDTA. Values are the averages of three samples ± SE. Different letters indicate that the means (between different Fe supplies) are different by the Tukey HSD test (*P* ≤ 0.05). Error bars may be too small to be visible in the figure. (JPEG 2 MB)

Below are the links to the authors’ original submitted files for images.

Authors’ original file for figure 1

Authors’ original file for figure 2

Authors’ original file for figure 3

Authors’ original file for figure 4

## Abbreviations

FM: Full maturity
GF: Grain filling
ICP-OES: Inductively coupled plasma optical emission spectroscopy
PE: Panicle exertion
PQ: Partition quotient.
