# Supplementary material for: Effects of different Fe supplies on mineral partitioning and remobilization during the reproductive development of rice (Oryza sativa L.)
Source: Rice (N Y). 2012 Sep 28;5:27. doi: 10.1186/1939-8433-5-27 (PMC4883723; doi:10.1186/1939-8433-5-27)
Supplement: Supplementary file 2 — Additional file 2: Mineral contents in rice organs. Contents of Fe, Zn, Cu, Mn, Mo, Ni, Ca, Mg, K and S in panicles, non-flag leaves, flag leaves, stems/sheaths and roots collected from rice plants supplied with different Fe concentrations at two reproductive growth stages. (DOCX 23 KB) [file 12284_2012_32_MOESM2_ESM.docx]

**Additional file 2.** **Mineral contents in rice organs.** Contents of Fe, Zn, Cu, Mn, Mo, Ni, Ca, Mg, K and S in panicles, non-flag leaves, flag leaves, stems/sheaths and roots collected from rice plants supplied with different Fe concentrations at two reproductive growth stages.

|  |  | **Content (μg plant^-1^)** | | | | | |
| --- | --- | --- | --- | --- | --- | --- | --- |
|  |  | **Grain Filling (GF)** | | | **Full Maturity (FM)** | | |
| **Organ** | **Element** | **5 µM Fe** | **20 µM Fe** | **200 µM Fe** | **5 µM Fe** | **20 µM Fe** | **200 µM Fe** |
| **Panicles** | **Fe** | 44.49 ± 0.39a | 59.67 ± 4.25a | 40.47 ± 13.02a | 61.23 ± 7.32a | 64.82 ± 12.96a | 95.75 ± 9.76a |
|  | **Zn** | 76.45 ± 3.20a | 84.71 ± 7.19a | 31.26 ± 3.94b | 127.24 ± 15.13a | 86.42 ± 16.31a | 73.16 ± 6.96a |
|  | **Cu** | 14.52 ± 0.51ab | 19.45 ± 1.67a | 9.65 ± 2.83b | 22.69 ± 2.57a | 18.35 ± 4.11a | 13.85 ± 1.92a |
|  | **Mn** | 91.45 ± 13.03a | 105.87 ± 14.02a | 27.99 ± 7.45b | 140.89 ± 19.55a | 115.75 ± 32.60a | 99.08 ± 14.18a |
|  | **Mo** | 2.05 ± 0.15a | 2.70 ± 0.43a | 1.25 ± 0.38a | 3.53 ± 0.54a | 2.67 ± 0.45ab | 1.89 ± 0.25b |
|  | **Ni** | 1.24 ± 0.02a | 0.76 ± 0.19ab | 0.24 ± 0.01b | 2.98 ± 0.48a | 0.56 ± 0.16b | 0.99 ± 0.14b |
|  | **Ca*** | 1.53 ± 0.10a | 1.82 ± 0.17a | 0.59 ± 0.15b | 1.98 ± 0.18a | 2.02 ± 0.66a | 1.75 ± 0.26a |
|  | **Mg*** | 3.22 ± 0.07ab | 3.74 ± 0.36a | 2.18 ± 0.48b | 4.16 ± 0.22a | 3.37 ± 0.75a | 2.99 ± 0.35a |
|  | **K*** | 19.57 ± 1.47a | 26.58 ± 3.33a | 13.29 ± 5.28a | 29.33 ± 3.30a | 30.02 ± 7.82a | 21.47 ± 2.91a |
|  | **S*** | 2.97 ± 0.09ab | 3.70 ± 0.30a | 1.95 ± 0.45b | 4.33 ± 0.43a | 3.85 ± 0.72a | 2.88 ± 0.31a |
| **Non-flag**  **leaves** | **Fe** | 122.02 ± 12.24a | 121.61 ± 12.69a | 145.75 ± 22.36a | 126.26 ± 18.20b | 125.71 ± 10.66b | 232.17 ± 29.72a |
|  | **Zn** | 32.45 ± 1.94a | 30.75 ± 3.61a | 30.92 ± 4.02a | 43.72 ± 7.08a | 39.84 ± 4.31a | 36.89 ± 4.06a |
|  | **Cu** | 21.50 ± 4.13a | 22.54 ± 2.33a | 16.41 ± 2.99a | 27.45 ± 4.30a | 22.94 ± 4.07a | 22.77 ± 4.05a |
|  | **Mn** | 204.38 ± 38.15a | 147.86 ± 34.90a | 139.51 ± 30.65a | 327.76 ± 58.77a | 151.10 ± 20.02b | 157.63 ± 25.79b |
|  | **Mo** | 14.10 ± 1.54a | 13.05 ± 1.51a | 10.43 ± 1.06a | 14.16 ± 2.03a | 15.44 ± 1.90a | 11.83 ± 1.50a |
|  | **Ni** | 1.31 ± 0.22a | 0.90 ± 0.09a | 0.82 ± 0.10a | 1.95 ± 0.23a | 1.02 ± 0.11b | 1.35 ± 0.12b |
|  | **Ca*** | 23.15 ± 3.37a | 22.34 ± 2.67a | 17.54 ± 3.16a | 28.87 ± 3.62a | 26.32 ± 4.17a | 25.47 ± 2.76a |
|  | **Mg*** | 10.91 ± 1.32a | 11.31 ± 1.32a | 9.63 ± 1.66a | 14.71 ± 2.12a | 12.89 ± 1.84a | 13.41 ± 1.68a |
|  | **K*** | 31.59 ± 2.94a | 45.28 ± 4.32a | 38.31 ± 2.49a | 46.80 ± 7.28a | 43.67 ± 4.62a | 43.37 ± 4.76a |
|  | **S*** | 7.35 ± 0.45a | 7.27 ± 0.53a | 6.23 ± 0.35a | 9.06 ± 0.74a | 10.04 ± 1.04a | 7.64 ± 0.65a |
| **Flag**  **leaves** | **Fe** | 19.06 ± 3.71a | 29.67 ± 2.24a | 24.05 ± 2.20a | 24.43 ± 1.87a | 23.75 ± 2.95a | 33.38 ± 4.00a |
|  | **Zn** | 8.92 ± 1.05b | 13.53 ± 0.71a | 10.09 ± 0.38b | 9.51 ± 1.41a | 9.67 ± 0.98a | 9.88 ± 0.82a |
|  | **Cu** | 4.21 ± 1.37a | 5.93 ± 0.33a | 3.32 ± 0.09a | 5.79 ± 0.94a | 3.52 ± 0.38a | 3.32 ± 0.48a |
|  | **Mn** | 14.27 ± 3.44ab | 36.50 ± 10.55a | 8.34 ± 1.80b | 62.88 ± 10.39a | 12.92 ± 2.47b | 16.38 ± 2.02b |
|  | **Mo** | 1.10 ± 0.31ab | 2.02 ± 0.25a | 0.83 ± 0.04b | 1.46 ± 0.12a | 1.09 ± 0.17ab | 0.89 ± 0.11b |
|  | **Ni** | 0.20 ± 0.03a | 0.22 ± 0.03a | 0.18 ± 0.00a | 0.25 ± 0.03a | 0.21 ± 0.03a | 0.21 ± 0.04a |
|  | **Ca*** | 1.71 ± 0.16b | 4.14 ± 0.41a | 1.42 ± 0.33b | 4.27 ± 0.44a | 2.74 ± 0.51b | 2.16 ± 0.28b |
|  | **Mg*** | 0.71 ± 0.03b | 1.85 ± 0.02a | 0.73 ± 0.09b | 1.64 ± 0.15a | 1.04 ± 0.15b | 0.96 ± 0.11b |
|  | **K*** | 7.95 ± 1.42b | 11.62 ± 0.79a | 8.00 ± 0.56b | 6.35 ± 0.89a | 6.29 ± 0.79a | 5.95 ± 0.82a |
|  | **S*** | 1.31 ± 0.13b | 2.16 ± 0.13a | 1.17 ± 0.05b | 1.69 ± 0.14a | 1.72 ± 0.23a | 1.10 ± 0.12a |
| **Stems/**  **Sheaths** | **Fe** | 126.33 ± 24.59a | 122.35 ± 10.82a | 123.94 ± 10.98a | 105.91 ± 15.08a | 114.33 ± 10.20a | 165.76 ± 21.50a |
|  | **Zn** | 160.41 ± 47.18a | 115.72 ± 26.27a | 58.38 ± 11.84a | 312.42 ± 57.57a | 104.45 ± 11.48b | 85.12 ± 11.27b |
|  | **Cu** | 34.88 ± 8.82a | 33.33 ± 3.09a | 20.90 ± 3.18a | 42.35 ± 5.86a | 36.65 ± 5.90a | 38.06 ± 6.95a |
|  | **Mn** | 171.70 ± 34.79a | 146.00 ± 14.31a | 98.54 ± 19.13a | 166.14 ± 24.45a | 120.53 ± 17.25a | 145.37 ± 14.81a |
|  | **Mo** | 10.16 ± 2.18a | 10.18 ± 1.38a | 6.70 ± 0.70a | 11.25 ± 1.83a | 11.40 ± 1.47a | 7.57 ± 1.05a |
|  | **Ni** | 2.43 ± 0.30a | 2.02 ± 0.26a | 1.64 ± 0.38a | 3.40 ± 0.13a | 1.13 ± 0.18b | 1.24 ± 0.14b |
|  | **Ca*** | 5.29 ± 0.95a | 6.09 ± 0.67a | 3.61 ± 0.83a | 6.49 ± 0.53a | 4.24 ± 0.65b | 5.07 ± 0.42ab |
|  | **Mg*** | 11.84 ± 2.21a | 12.56 ± 0.95a | 8.90 ± 1.30a | 11.03 ± 1.57a | 12.14 ± 1.60a | 10.70 ± 1.22a |
|  | **K*** | 98.19 ± 15.83b | 157.19 ± 9.27a | 115.20 ± 10.84ab | 141.03 ± 15.29a | 146.12 ± 18.17a | 131.76 ± 11.39a |
|  | **S*** | 10.03 ± 1.72a | 10.10 ± 0.87a | 7.49 ± 0.80a | 10.40 ± 1.40a | 9.57 ± 1.18a | 8.90 ± 0.93a |
| **Roots** | **Fe** | 680.30 ± 173.54a | 833.67 ± 80.51a | 915.57 ± 224.27a | 923.19 ± 151.02b | 563.42 ± 99.14b | 2,170.42 ± 211.02a |
|  | **Zn** | 86.31 ± 26.54a | 34.70 ± 4.61ab | 10.87 ± 0.65b | 109.73 ± 16.48a | 29.88 ± 5.69b | 23.96 ± 3.51b |
|  | **Cu** | 24.03 ± 6.52a | 20.58 ± 5.21a | 4.76 ± 0.06b | 93.83 ± 20.55a | 14.97 ± 4.61b | 10.76 ± 2.41b |
|  | **Mn** | 14.36 ± 3.52a | 24.89 ± 6.03a | 12.99 ± 4.04a | 17.65 ± 3.02a | 19.28 ± 2.83a | 26.02 ± 3.68a |
|  | **Mo** | 2.35 ± 0.52a | 3.17 ± 0.66a | 1.83 ± 0.90a | 3.96 ± 0.73a | 3.79 ± 1.06a | 1.54 ± 0.27a |
|  | **Ni** | 2.26 ± 0.56a | 0.76 ± 0.12b | 0.35 ± 0.11b | 6.62 ± 1.15a | 0.68 ± 0.15b | 1.43 ± 0.15b |
|  | **Ca*** | 7.23 ± 1.27b | 24.52 ± 2.87a | 18.04 ± 4.18ab | 5.62 ± 0.79c | 25.19 ± 4.71a | 14.20 ± 1.88b |
|  | **Mg*** | 0.39 ± 0.12a | 0.50 ± 0.06a | 0.42 ± 0.16a | 0.53 ± 0.09a | 0.51 ± 0.11a | 0.40 ± 0.08a |
|  | **K*** | 5.94 ± 1.57a | 9.80 ± 2.60a | 5.50 ± 1.51a | 12.96 ± 2.55a | 10.29 ± 2.98a | 4.90 ± 1.20a |
|  | **S*** | 1.90 ± 0.69a | 1.98 ± 0.34a | 1.42 ± 0.59a | 2.72 ± 0.46a | 2.23 ± 0.55a | 1.78 ± 0.48a |

Data are mean ± standard error. *n* = 4 for the grain filling (GF) stage and *n* = 8 for the full maturity (FM) stage. *Contents of Ca, Mg, K and S are presented as mg plant^-1^. Mean values (between different Fe supplies) indicated by different letters are different by the Tukey HSD test (*P* ≤ 0.05).
