# Supplementary figures and images for: Effects of different Fe supplies on mineral partitioning and remobilization during the reproductive development of rice (Oryza sativa L.)
Source: Rice (N Y). 2012 Sep 28;5:27. doi: 10.1186/1939-8433-5-27 (PMC4883723; doi:10.1186/1939-8433-5-27)

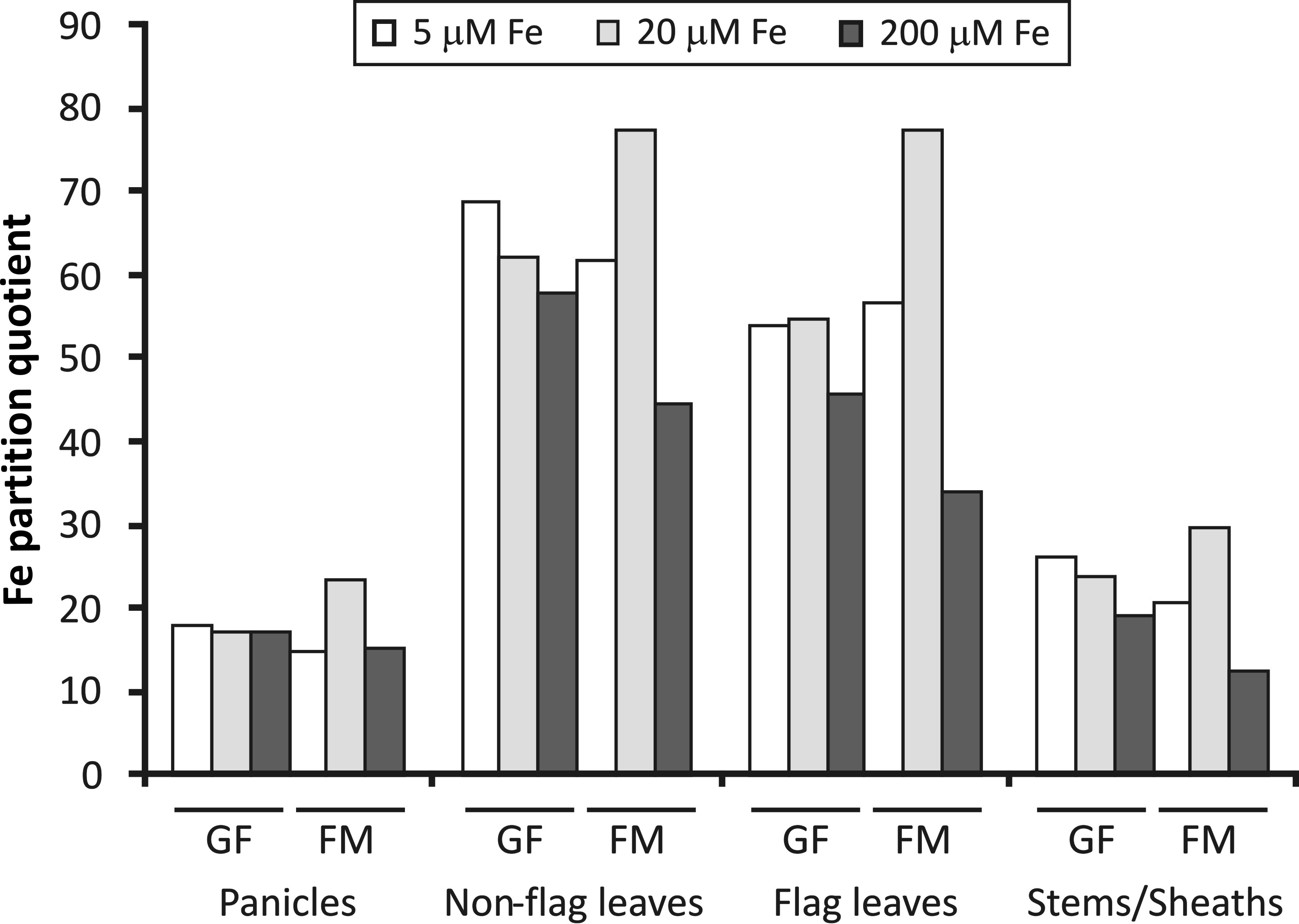

Supplement: Supplementary file 5 — Additional file 5: Iron partition quotient (PQ) in rice organs. Iron partition quotient in panicles, non-flag leaves, flag leaves and stems/sheaths during grain filling (GF) and full maturity (FM) stages of rice plants cultivated with 5, 20 or 200 μM of Fe(III)-HEDTA. (JPEG 388 kb) (JPEG 388 KB) [file 12284_2012_32_MOESM5_ESM.jpeg]

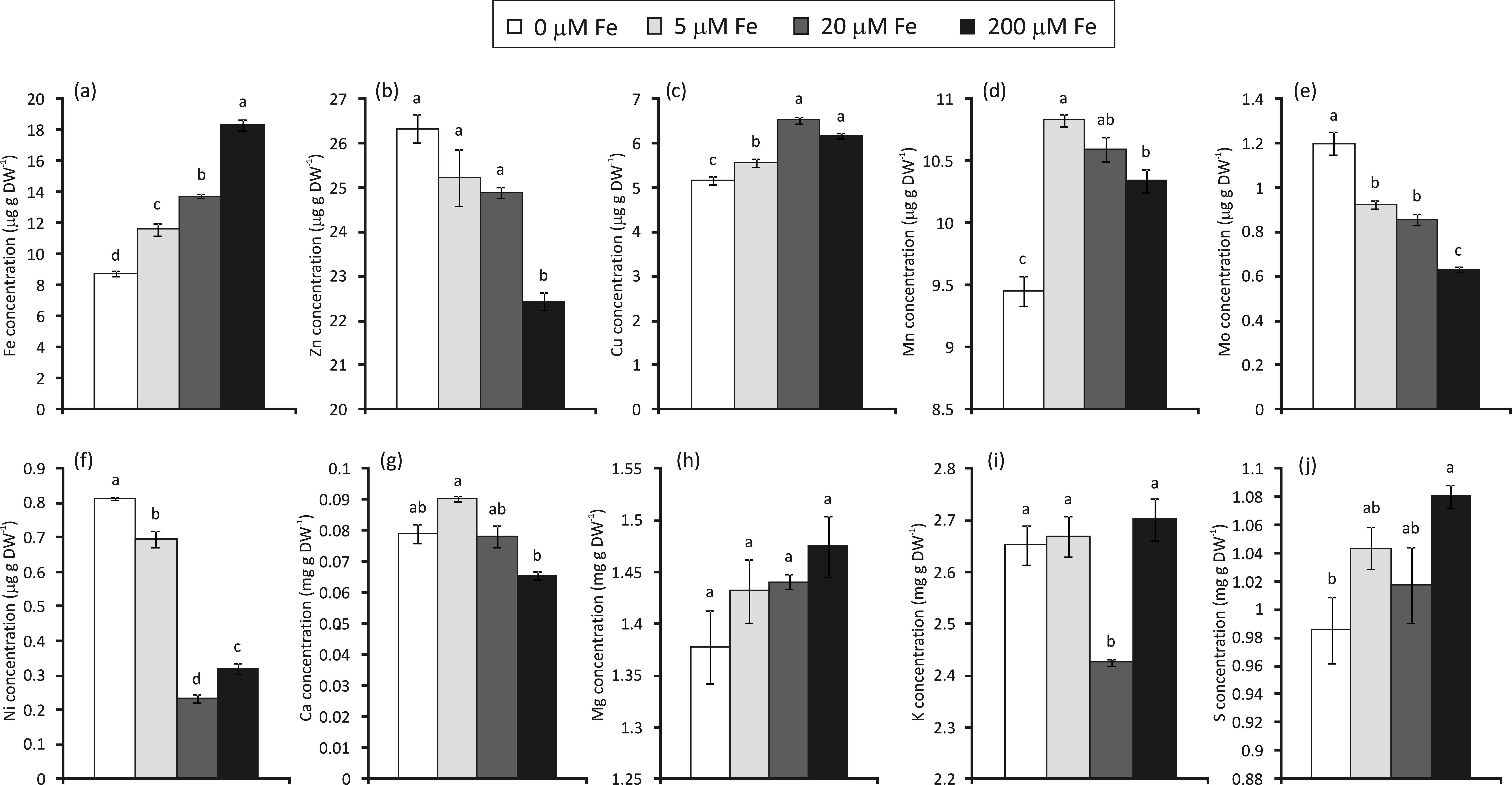

Supplement: Supplementary file 6 — Additional file 6: Mineral concentrations in rice seeds. Fe, Zn, Cu, Mn, Mo, Ni, Ca, Mg, K and S concentrations in de-husked unpolished seeds collected during full maturity stage of rice plants cultivated with 0, 5, 20 or 200 μM of Fe(III)-HEDTA. Values are the averages of three samples ± SE. Different letters indicate that the means (between different Fe supplies) are different by the Tukey HSD test (P ≤ 0.05). Error bars may be too small to be visible in the figure. (JPEG 2 MB) [file 12284_2012_32_MOESM6_ESM.jpeg]

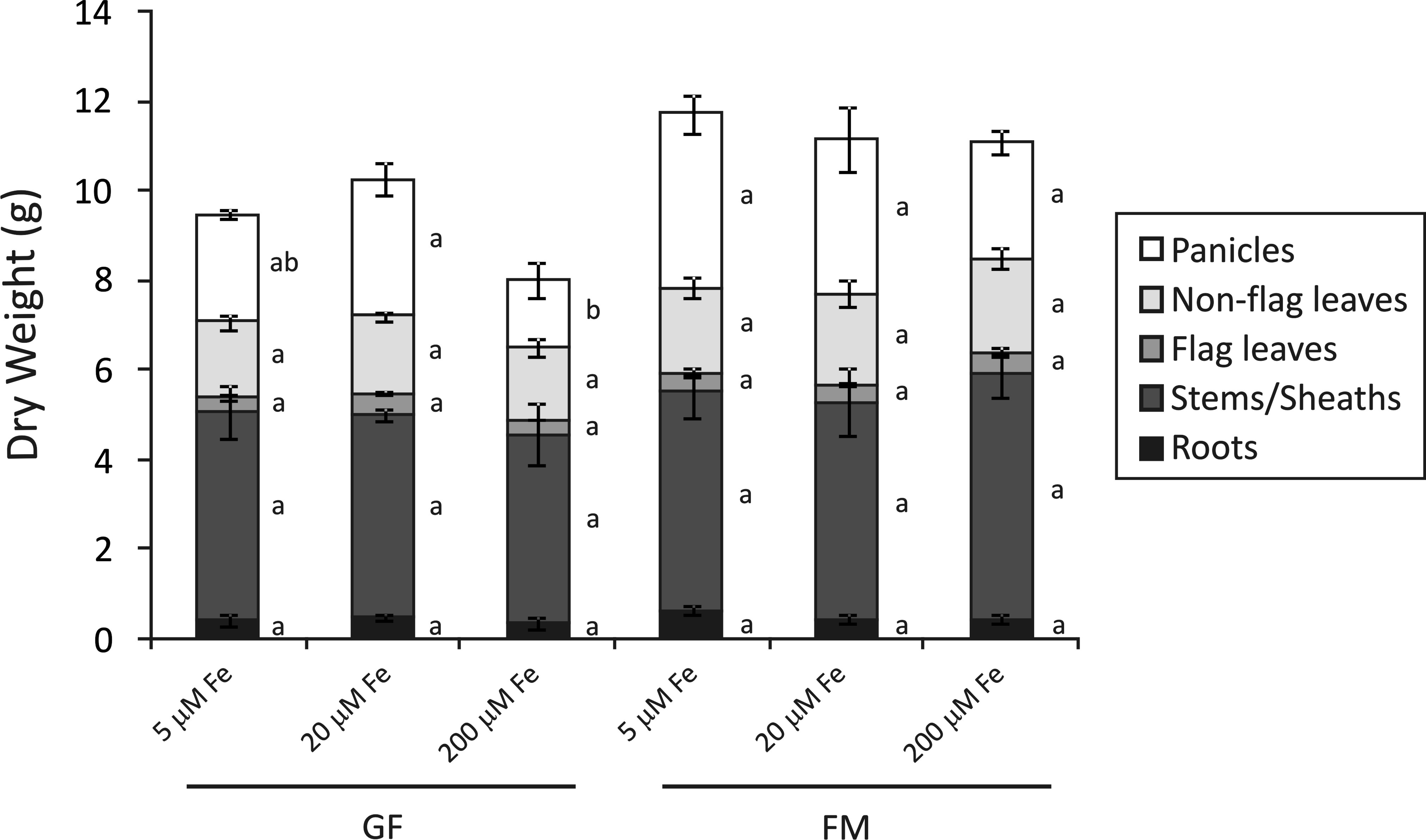

Supplement: Supplementary file 7 — Authors’ original file for figure 1 [file 12284_2012_32_MOESM7_ESM.jpeg]

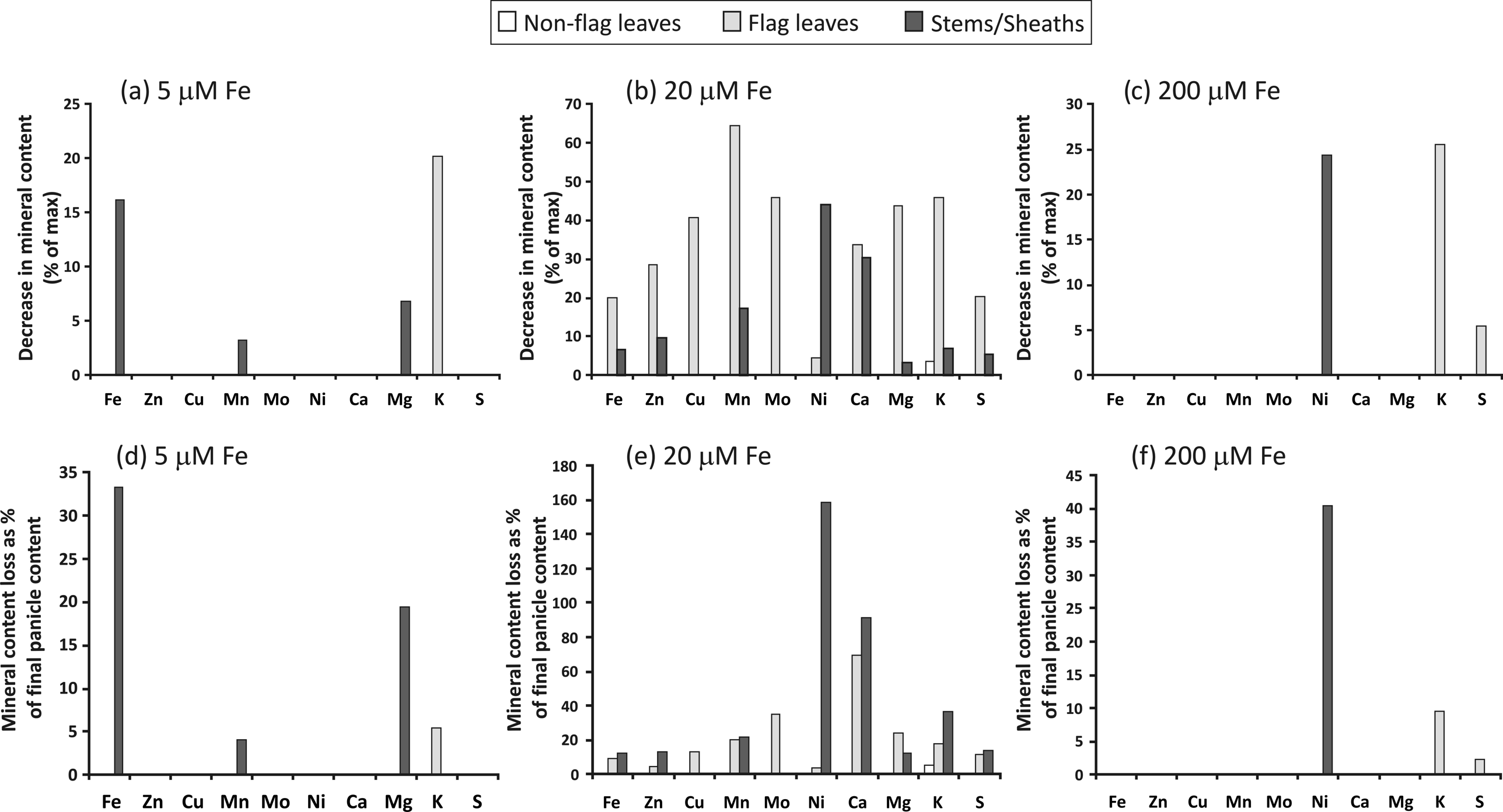

Supplement: Supplementary file 10 — Authors’ original file for figure 4 [file 12284_2012_32_MOESM10_ESM.jpeg]
